# Bioprinting neural tissue to decode Sandhoff disease: promise and barriers

**DOI:** 10.1097/MS9.0000000000004131

**Published:** 2025-10-28

**Authors:** Syed Haibat Ullah, Muhammad Waqas, Mahnoor Fatima, Muhammad Talha, Ubaid Ur Rehman

**Affiliations:** a Department of Medicine, Bolan Medical College, Quetta, Pakistan; b Department of Medicine, Gomal Medical College, Dera Ismail Khan, Pakistan; c Department of Medicine, Shaheed Ziaur Rahman Medical College, Rajshahi Medical University, Bogura, Bangladesh; d Department of Medicine, King Edward Medical University, Lahore, Pakistan

*To the Editor,*

Sandhoff disease is a rare lysosomal storage disorder inherited in an autosomal recessive manner. It results from mutations in the HEXB gene, which cause deficiency of both β-hexosaminidase A and B enzymes, leading to the build-up of GM2 ganglioside in nerve cells. The condition has a broad clinical spectrum: the infantile form typically presents within the first months of life, characterized by developmental regression, seizures, low muscle tone, and the characteristic cherry-red spot in the retina. In contrast, adult forms progress more slowly, often accompanied by motor impairment, cognitive decline, and occasionally psychiatric features. Currently, management is primarily supportive, focusing on controlling seizures, easing muscle stiffness, and maintaining adequate nutrition. These measures can help improve comfort and quality of life, but no curative or disease-modifying therapy exists, underscoring the urgent need for new treatment approaches^[1]^. This is in line with the TITAN Guidelines on the need for transparency in AI use in healthcare^[2]^.

In recent years, three-dimensional (3D) bioprinting of neural tissues has emerged as a promising tool for studying neurodegenerative and lysosomal storage disorders, including Sandhoff disease. Although specific applications for this condition are still limited, encouraging results have been reported in related areas^[3]^. For example, bioprinted neural tissues created from mesenchymal stem cells using fibrin-based bioinks have shown expression of neuronal markers, differentiation toward dopaminergic lineages, and immature electrophysiological activity. Similarly, patient-derived induced pluripotent stem cell (hiPSC) constructs for Alzheimer’s disease have demonstrated better cell survival, enhanced maturation, and functional signaling when optimized bioinks and microsphere supports were used. These findings suggest that 3D bioprinting may offer valuable insights and potential avenues for developing therapies in the future^[4]^.

Gene therapy is a promising route for treating Sandhoff disease. Preclinical trials using adeno-associated viral vectors have improved survival in animal models, and early clinical studies in GM2 gangliosidosis have reported encouraging biomarker changes^[5]^. In this landscape, 3D bioprinted neural tissue provides a unique research platform. These engineered constructs replicate essential features of neural organization, including cellular diversity and extracellular interactions, which are absent in standard culture systems. Combined with hiPSCs, they offer opportunities to investigate lysosomal dysfunction, demyelination, and neuroinflammation with greater precision. With further advances such as vascularized printing and matrix-enriched bioinks, 3D bioprinted tissue may one day extend beyond disease modeling to therapeutic applications, including grafting support and neural repair in lysosomal storage disorders like Sandhoff disease^[6]^.

Although 3D bioprinting presents a new platform for disease modeling, there are still major barriers that limit its use in Sandhoff disease. Current bioprinted designs are limited in terms of their capacity to accurately replicate the complex neural microenvironment in vivo because they frequently lack vascularization and long-term stability. This makes it more difficult to translate research results into treatments that have clinical value and demonstrates that the technology is currently still in its early stages of development rather than being prepared for therapeutic use. Furthermore, the majority of bioprinted neural tissue has difficulty in achieving full maturation and functional integration, which diminishes their ability to mimic prolonged disease progression. Also, the high cost required for a bioprinting platform may limit accessibility and use in rare diseases such as Sandhoff disease^[7]^.

3D bioprinted neural tissue is becoming a useful approach to study the complex changes that occur in GM2 gangliosidoses, such as Sandhoff disease. Studies using stem cell-based constructs^[3]^ suggest that these models can show key problems like lysosomal dysfunction and inflammation in the nervous system more clearly than older methods. At the same time, advances in intracerebral gene therapy for GM2 gangliosidosis^[5]^ show how bioprinting might work alongside new treatment strategies. Still, issues such as a lack of blood vessel networks and weak structural stability mean the technology is at an early stage and not yet ready for direct clinical use.

In conclusion, 3D bioprinted neural tissue has great potential as a model for studying and treating Sandhoff disease. However, its application is mostly at the preclinical level. The potential of such disease models and associated organoids offers the possibility to dissect pathomechanisms and test treatment, but limitations such as missing vascularization, certain restrictions regarding structural stability in vitro, as well as the current lack of Sandhoff-specific models, indicate that this field is still emerging. Follow-up studies should focus on the construction of patient-specific bio-printed models for Sandhoff disease, which will offer a more realistic platform to study lysosomal function and therapeutic responses. Moreover, the crossover of bioprinting with novel modalities like gene therapy and substrate reduction strategies has the potential to facilitate the journey toward personalized therapies.

## Data Availability

Not applicable to this study.

## Research registration unique identifying number (UIN)

No human subjects were involved.
